# Mealworm (*Tenebrio molitor* Larvae) as an Alternative Protein Source for Monogastric Animal: A Review

**DOI:** 10.3390/ani10112068

**Published:** 2020-11-08

**Authors:** Jinsu Hong, Taehee Han, Yoo Yong Kim

**Affiliations:** 1 Department of Animal Science, South Dakota State University, Brookings, SD 57007, USA; jinsu.hong@sdstate.edu; 2 Department of Production Animal Medicine, Faculty of Veterinary Medicine, University of Helsinki, 00014 Helsinki, Finland; taehee.han@helsinki.fi; 3 Department of Agricultural Biotechnology, and Research Institute of Agriculture and Life Sciences, Seoul National University, Seoul 08826, Korea

**Keywords:** alternative protein, *Tenebrio molitor* larvae, mealworm, pig, poultry

## Abstract

**Simple Summary:**

*Tenebrio molitor* (*T. molitor*) larvae, known as mealworm, have been considered a good protein source for monogastric animals. They have a high quantity and quality of protein content and amino acid profile. The inclusion of *T. molitor* larvae in broiler diets improved the growth performance without having negative effects on carcass traits and blood profiles in broiler chickens, or had no influence on the growth performance and carcass yield of broiler chickens. The supplementation of *T. molitor* larvae improved the growth performance and protein utilization of weaning pigs. Furthermore, the replacement of fishmeal with *T. molitor* larvae resulted in no difference in the growth performance and nutrient digestibility of weaning pigs. However, there are some challenges regarding biosafety, consumer’s acceptance, and price for the use of *T. moiltor* larvae in animal feed. Consequently, *T. molitor* larvae could be used as an alternative or sustainable protein source in monogastric animal feed.

**Abstract:**

Edible insects have been used as an alternative protein source for food and animal feed, and the market size for edible insects has increased. *Tenebrio molitor* larvae, also known as mealworm and yellow mealworm, are considered a good protein source with nutritional value, digestibility, flavor, and a functional ability. Additionally, they are easy to breed and feed for having a stable protein content, regardless of their diets. Therefore, *T. molitor* larvae have been produced industrially as feed for pets, zoo animals, and even for production animals. To maintain the nutrient composition and safety of *T. molitor* larvae, slaughtering (heating or freezing) and post-slaughtering (drying and grinding) procedures should be improved for animal feed. *T. molitor* larvae are also processed with defatting or hydrolysis before grinding. They have a high quality and quantity of protein and amino acid profile, so are considered a highly sustainable protein source for replacing soybean meal or fishmeal. *T. molitor* has a chitin in its cuticle, which is an indigestible fiber with positive effects on the immune system. In studies of poultry, the supplementation of *T. molitor* larvae improved the growth performance of broiler chickens, without having negative effects on carcass traits, whereas some studies have reported that there were no significant differences in the growth performance and carcass yield of broiler chickens. In studies of swine, the supplementation of *T. molitor* larvae improved the growth performance and protein utilization of weaning pigs. Furthermore, 10% of *T. molitor* larvae showed greater amino acid digestibility than conventional animal proteins in growing pigs. However, there are some challenges regarding the biosafety, consumer’s acceptance, and price for the use of *T. moiltor* larvae in animal feed. Consequently, *T. molitor* larvae could be used as an alternative or sustainable protein source in monogastric animal feed with a consideration of the nutritional values, biosafety, consumer’s acceptance, and market price of *T. molitor* larvae products.

## 1. Introduction

The International Feed Industry Federation (IFIF) reported that the world population will be reach more than 10 billion people by 2050 [1]. Furthermore, the expanded population is expected to consume almost double the amount of animal protein [2]. In the case of pork and poultry meat, the expected growth of consumption from 2010 to 2050 is 105% and 173%, respectively [2]. This implies that animal feed will be a critical component of the integrated food chain in the future. Meyer-Rochow [3] suggested as far back as in 1975 that using edible insects as food and feed may ease global food shortages. The Food and Agriculture Organization (FAO) stresses the importance of finding alternatives to conventional animal feed because of its limited amount [2]. Currently, the major protein sources in monogastric animals (e.g., poultry and swine) diets are fishmeal, processed animal protein, milk by-product, soybean meal (SBM), rapeseed meal, and canola meal. The price of these conventional protein sources, however, has increased because of the limited production and the competition between humans and animals [4]. Therefore, considering the increasing world population and the price of conventional protein sources, it is important to find an alternative protein source in the future.

In the last few decades, edible insects (entomophagy) have been used as an alternative nutritional source for both food and animal feed. This is because they are edible proteins source for both humans and animals [5] and the percentage of edible parts is nearly 100% [6,7]. Moreover, the nutritional value of insects is much better than that of plants in terms of higher protein, essential amino acids, vitamin, and mineral contents (this will be discussed below). When they are fed to monogastric animals (e.g., poultry and swine), the growth performance and digestibility seem to improve compared to other protein sources [5]. Not only because of their nutritional value, but also because of their environmental impact, the insects will play a major role in future protein sources for animal feed. For instance, insects have lower greenhouse gas production [8], use of water [9], and use of arable land [6] than other animal species (e.g., pig, cattle, and poultry). The production of insects for food and feed is sustainable [10]. For instance, *T. molitor* is widely used for the biodegradation of organic waste to proteins [11]. Furthermore, such specimens can degrade plastic waste to proteins because of their unique gut microbiome [12,13].

Edible insects as animal feed ingredients have been studied by many researchers. In particular, insects as an alternative protein source in broiler chicken [14,15,16,17] and pig [18,19,20] diets have been widely investigated. It seems that the demand for edible insects for feed ingredients will increase in the near future. Accordingly, many companies have started the large-scale production of insects for food and feed worldwide (e.g., France and China). Therefore, the price and usage of an insect as a feed ingredient will become reasonable and broaden, respectively.

In this review, we focused on *T. molitor* larvae as an alternative protein source for monogastric animals because they have recently been proven to be a proper feed ingredient for poultry and swine diets by many researchers. We aimed to provide their nutritional value, their impact on the growth performance, and possible challenges when they are fed to monogastric animals.

## 2. *Tenebrio molitor* Larvae

Among insect sources, *T. molitor* larvae are known to have a good nutritional value, such as protein and fat content [7,14,15,21,22], digestibility [19,20], flavor [18,23], and functional ability (e.g., chitin and antimicrobial peptides, i.e., AMPs) [24,25,26,27,28]. They are easy to breed and raise and have a stable protein content, regardless of their diets [29], which implies that *T. molitor* larvae can be produced stably. For this reason, they have been produced industrially as feed for pets, zoo animals, and even for production animals, such as fish, pigs, and poultry [14,18].

*T. molitor* is a pest of flour, grain, and food and is distributed worldwide [14]. It is a species of darkling beetle and has four life stages: egg, larva, pupa, and adult. Female *T. molitor* specimens lay approximately 500 eggs, which hatch after 3–9 days and become larvae at 25 °C [30,31,32]. The larva stage lasts 1–8 months and has a light yellow-brown color [30,31]. The pupal stage lasts 5–28 days at 18°C [30,31,32] and the adult stage lasts 2–3 months [30]. The size of the larva is usually about 2.0–3.5 cm or more [30,33] and that of adults is approximately 1 cm [30]. This adult is one of the biggest beetles [32]. It is omnivorous and can eat plant and animal products, such as meat and feathers, but the diet should be formulated to contain 20% protein [14]. In a commercial situation, these specimens are mainly fed on cereal bran or flour (wheat, oats, and maize), and fruits and vegetables are supplemented for moisture. The protein sources for *T. molitor* are soybean flour, skimmed milk powder, and yeast [11,33]. Compositions of *T. molitor* seem to be affected by diets. Some studies have observed that the composition of a cereal-based diet did not alter the major nutritional composition (e.g., crude protein, crude fat, and moisture) of *T. molitor* larvae [14,29,34], whereas the fatty acid (e.g., caprylic acid) composition seemed to be affected by an unsaturated fatty acid-enriched diet [34]. Higher protein and lower fat contents were observed when they were fed plant waste compared to a cereal-based diet [31]. A recent study also showed that the composition of *T. molitor* larvae was affected by the type of diet [35]. This implies that we should identify the optimal diet for *T. molitor* to obtain a stable and better nutritional composition for food and feed. *T. molitor* has an efficient growth rate compared to other production animals. *T. molitor* has more than 3 for feed conversion ratio (FCR) for fresh weight [29,36], while poultry meat and pork have 2.3 and 4.0 for FCR, respectively [37]. Considering the edible part of insect is almost 100%, FCR of *T. molitor* seems to be higher than that of total edible parts of poultry meat and pork (1.7 and 2.3, respectively; [37]). Bjørge et al. [38] also observed a 16.6% growth rate (growth rate/day, %) for *T. molitor* at an optimal temperature, which is higher than that of poultry. Ghaly and Alkoaik [30] suggested that the early larva stage (about 100–120 mg of weight) is the most efficient because the growth rate decreased after that stage. The type of diet also affects the growth and reproduction performance of *T. molitor* larvae [35]. In the study of Rumbos et al. [35], a better reproductive performance and larvae development were observed in amylaceous substrates, such as wheat bran and white flour. Therefore, optimized feeding for *T. molitor* larvae represents one option for providing a proper nutritional composition and ensuring efficient production.

As the interest in *T. molitor* larvae has increased as a future food and feed ingredient, there have been many efforts to find the optimal rearing condition [38,39]. In general, they are commercially available with a low level of technology for the biological control industry [39]. This may allow *T. molitor* larvae to become an efficient protein source. Furthermore, the commercial production market has also broadened. Based on recent studies, it seems that commercial *T. molitor* larvae are available in China [15,40,41], the USA [13], France [42], and Spain [43]. Therefore, *T. molitor* larvae may become a proper alternative protein source in the future.

## 3. Processing of *Tenebrio molitor* Larvae

**Figure 1:**
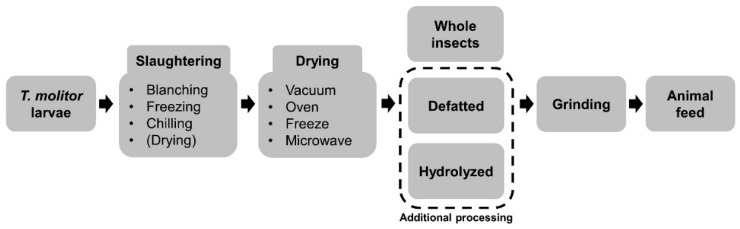
Scheme of *Tenebrio molitor* larvae processing for animal feed.

The International Platform of Insects for Food and Feed (IPIFF) [44] reported that insects are typically processed by slaughtering (heating or freezing) and post-slaughtering (drying and grinding) procedures for animal feed (Figure 1). These procedures are important not only for ensuring safety, but also for maintaining the nutrient composition. The slaughtering process includes blanching, freezing, chilling, and drying. This allows the long-term storage and transport of *T. molitor* larvae. Many researchers have attempted to find the optimal method for optimizing both the safety and nutritional value. Vandeweyer et al. [45] suggested blanching before chilling or drying because blanching enables vegetative cells to be killed and prevents microbial growth during storage. After slaughtering, drying is important because of the high moisture content of the *T. molitor* larvae (approximately 68%). This high moisture content might cause enzymatic or non-enzymatic degradation and microbiological spoilage [46]. Therefore, a 4 to 5% moisture content is recommended to avoid possible problems [47]. The major methods employed for drying are oven drying [46], vacuum drying [46], freeze-drying [46,48], and microwave drying [45]. Kröncke et al. [46] reported that different drying types did not result in hugely different nutritional values for the *T. molitor* larvae. Moreover, all of those drying types resulted in low water activity in *T. molitor* larvae and this content does not allow microbiological growth [49]. In the study of Kröncke et al. [46], on the other hand, oven drying had the shortest processing time and lowest energy consumption. Therefore, blanching and oven drying might be the most efficient way to process *T. molitor* larvae. After drying, the specimens are usually finely ground to the same particle size as other feed ingredients.

Before grinding, *T. molitor* larvae may undergo additional processing steps such as defatting or hydrolysis (Figure 1). *T. molitor* larvae have been supplemented as whole (full-fat) ground [14,18,50], defatted ground [20,51,52,53], or hydrolyzed ground [20] feed ingredients. Defatting is important for ensuring longer storage and processing. This is because full-fat *T. molitor* larvae contain a high fat (25–35%) and fatty acid composition (10–30%) and those contents make them susceptible to lipid oxidation during drying and storage [54,55]. Defatting can be conducted by either high pressing [42], or using an organic solvent [56] and supercritical CO_2_ [57,58]. A recent study supplemented hydrolyzed *T. molitor* larvae in pig diet to reduce the possible anti-nutritive factors [20]. They observed that hydrolyzed *T. molitor* larvae improved the ileal digestibility of pigs. Extracting and purifying the protein or fat from *T. molitor* larvae have also been made possible as the processing technique has evolved [48,56]. Until now, they have been used as food ingredients for humans with nutritional and functional aspects [59]. However, extracted protein or fat from *T. molitor* larvae could also be used for animal feed and this needs to be studied. The functional properties of protein extracted from *T. molitor* larvae seem to be affected by the processing temperature and time [56,60]. Therefore, suitable and efficient processing steps of *T. molitor* larvae for animal feed ingredients should be established in the future.

## 4. Nutritional Value of *Tenebrio molitor* Larvae

*T. molitor* larvae have been used as feed ingredients for animal diets. This is because the production efficiency of *T. molitor* larvae is higher than that of the adults. The ingredient of *T. molitor* larvae is produced by drying and grinding and larvae meal is produced from a by-product of oil extraction for *T. molitor* larvae.

The nutritional value of *T. molitor* larvae or larvae meal is presented in Table 1. The crude protein (CP) content of *T. molitor* larvae shows an average of 52.4% and ranges from 47.0% to 60.2%, which is greater than that of conventional SBM (49.4%, [61]) and less than that of fishmeal (67.5%, [61]). The crude protein values for *T. molitor* larvae or larvae meal presented in Table 1 were analyzed by combustion (Dumas) method [28], Randall method [62,63], Kjeldahl method [15,16,19,54,64,65], and elemental analysis method [65]. It should be noted that protein contents for insect are often overestimated with the use of nitrogen-to-protein conversion factor (*k_p_*) 6.25 [66]. The chitin exoskeleton, a polysaccharide of glucosamine and *N*-acetylgluocsamine, is considered non-protein nitrogen and indigestible protein. Recent studies have suggested that nitrogen-to-protein conversion factor for *T. molitor* larvae is 4.74 [67], 4.75 [68], or 5.41 [65]. With regard to the application of *k_p_* 5.41 for *T. molitor* larvae [65], the CP content of *T. molitor* larvae shows an average 47.2% and ranges from 43.9% to 51.0%, which is similar to that of conventional SBM (49.4%, [61]) and less than that of fishmeal (67.5%, [61]). The *T. molitor* larvae contain an average of 30.8% (range from 19.1% to 36.7%) of crude fat and this varies depending on the processing method. The crude fat values for *T. molitor* larvae were greater than those for SBM (1.4%) and fishmeal (10.4%) that were reported by national research council (NRC) [61]. However, the average content of crude ash (4.2%) for *T. molitor* larvae is lower than those for SBM (7.2%, [61]) and fishmeal (17.2%, [61]) and ranges from 2.65% to 6.99%.

Whole insects contain a variable amount of fiber as measured by crude fiber, acid detergent fiber (ADF), and neutral detergent fiber (NDF) [69,70,71,72]. The content of fiber in *T. molitor* larvae originates from their cuticles. The crude fiber content of *T. molitor* larvae exhibits an average 7.43% and ranges from 4.19% to 22.35%. The average crude fiber content of *T. molitor* larvae is similar to that of SBM (7.43%) and higher than that of fishmeal (0.26%) [61]. The ADF value for *T. molitor* larvae is 7.66% [16] and the NDF value for *T. molitor* larvae is 17.4% [72]. The ADF content of *T. molitor* larvae is similar to that of SBM (7.66% vs. 7.50%, respectively) [16,61], but the NDF value (17.4%) for *T. molitor* larvae is higher than that for SBM (17.4% vs. 11.06%, respectively) [61,72]. The fiber in insects represents chitin, which is in a complex matrix with cuticular proteins, lipids, and other compounds [73]. Since chitin (linear polymer of β-(1-4)N-acetyl-D-glucosamine units) has a similar molecular structure to that of cellulose (linear polymer of β-(1-4)D-glucopyranose units), ADF values adjusted for the amino acid content can be recommended for estimating chitin contents in insects [74].

*T. molitor* larvae have a high quality and quantity of protein and amino acid profile, so are considered a highly sustainable protein source alternative to SBM or fishmeal. The amino acid profile of *T. molitor* larvae is presented in Table 2. The Leu, Val, and Lys are the most abundant indispensable amino acids in *T. molitor* larvae, whereas His, Met, and Trp are the least abundant. The Lys content for *T. molitor* larvae ranges from 1.58% to 5.76%, and the Met content for *T. molitor* larvae ranges from 0.52% to 2.20%. Additionally, the Thr values for *T. molitor* larvae ranges from 1.57% to 4.29%, and the Trp values for *T. molitor* larvae ranges from 0.02% to 1.86%. The *T. molitor* larvae have greater contents of Lys, Met, Thr, Trp, Val, and Ile compared to those of SBM [61]. Although the Lys, Met, and Thr contents for *T. molitor* larvae are lower than those for fishmeal, the Trp, Val, and Ile contents are greater than those for fishmeal [61].

The fatty acid composition of *T. molitor* larvae is presented in Table 3. Regarding the saturated fatty acids (SFA) of *T. molitor* larvae, myristic acid (C14:0) ranges from 2.12% to 5.21%, palmitic acid (C16:0) ranges from 9.33% to 17.21%, and stearic acid (C18:0) ranges from 0.26% to 3.06%. Palmitoleic acid (C16:1) ranges from 9.33% to 17.24%, oleic acid (C18:1n9) ranges from 40.78% to 49.71%, linoleic acid (C18:2n6) ranges from 24.19% to 35.58%, linolenic acid (C18:3n3) ranges from 0.35% to 2.27%, γ-linoleic acid (C18:3n6) ranges from 0.03% to 1.85%, and eicosenoic acid (C20:1n9) ranges from 0.06% to 0.39%, which were reported as the unsaturated fatty acids (UFA) in *T. molitor* larvae. The SFA and UFA for *T. molitor* larvae range from 22.3% to 23.3% and from 77.7% to 79.0%, respectively [62,63]. The *T. molitor* larvae have similar composition of UFA when compared to poultry meal and fishmeal [19]. Furthermore, *T. molitor* larvae contain essential polyunsaturated fatty acids (PUFAs), such as omega 3 and 6 acids. A total of 46.1 to 47.3 g/100 g of omega 3 acid and 31.1 to 31.6 g/100 g of omega 6 acid were detected in *T. molitor* larvae [62,63].

The mineral content for *T. molitor* larvae is presented in Table 4. The calcium values for *T. molitor* larvae range from 0.04% to 0.50%, and the phosphorus values for *T. molitor* larvae range from 0.70% to 1.04%. Additionally, the sodium values for *T. molitor* larvae range from 0.21% to 0.36%, and the potassium values for *T. molitor* larvae range from 0.85% to 1.12%. The iron values for *T. molitor* larvae range from 63.0 to 100.0 mg/kg, and the zinc values for *T. molitor* larvae range from 102.0 to 117.4 mg/kg. Moreover, the copper values for *T. molitor* larvae range from 12.3 to 20.0 mg/kg.

Chitin can be hardened and transformed into an exoskeleton [78]. It is considered an indigestible fiber with positive effects on the functioning of the immune system [79,80]. Furthermore, it can improve the immune status and performance of animals [81,82]. The composition and amount of chitin in insects vary with the species and developmental stages. Most of the chitin remains in exuviate, which has a high percentage of chitin of about 18.35%. An adult is produced by metamorphosis 15–20 times from the egg for exuviate accumulation. The *T. molitor* larva contains the lowest content of chitin in comparison to other types. Adámková et al. [25] found that *T. molitor* larvae contained 13 g/100 g of chitin and *T. molitor* pupa contained 12 g/100 g of chitin. The chitin contents of *T. molitor* larvae vary between studies. The chitin content of *T. molitor* larvae has an average of 6.41%, ranging from 4.92% to 13.0% [25,26,28,75,83,84]. Finke [74] estimated that the chitin content in *T. molitor* adults was 137.2 mg/kg on a dry matter (DM) basis. Chitosan, which is considered as a substance for biomedical use, is produced from chitin by deacetylation [26]. *T. molitor* larvae were shown to contain 11.56 mg/g of chitosan [18], which implies that mealworm larvae can be used as functional food or feed.

## 5. *Tenebrio molitor* Larvae in Monogastric Animal Nutrition

### 5.1. Poultry

The effects of *T. molitor* larvae supplementation in broiler diets are presented in Table 5. The inclusion of *T. molitor* larvae from 0% to 0.3% in diets fed to broiler chickens (0 to 42 d) improved the body weight gain (BWG), feed conversion ratio (FCR), and dressing rate of broiler chickens [75]. Similarly, Benzertiha et al. [28] reported that the inclusion of full-fat *T. molitor* larvae meal from 0% to 0.3% in broiler diets increased the BWG, feed intake (FI), and FCR, and decreased weight of bursa of Fabricius. They also reported that the dietary inclusion of full-fat *T. molitor* larvae meal at 0.3% decreased the serum IgM and blood non-esterified fatty acids, and increased the serum IL-2 and TNF-α. Increasing the inclusion level of full-fat *T. molitor* larvae meal from 0% to 15% in diets fed to broiler chickens (0 to 53 day) increased the body weight (BW) at 12 and 25 day, daily feed intake, and FCR, but did not affect the weights of the carcass, breast, and thigh in broiler chickens [17]. Sedgh-Gooya et al. [64] reported that the inclusion of *T. molitor* larvae meal from 0 to 5% in broiler diets increased the BWG for 0 to 10 day, whereas it did not affect the daily FI, FCR, carcass yield and organ weights. Elahi et al. [85] reported that increasing the inclusion of *T. molitor* larvae meal in broiler diets increased the BW, BWG, and FCR for 0 to 21 day, but did not affect the growth performance for 21 to 42 d and 0 to 42 day. They also reported that the inclusion level of *T. molitor* larvae meal from 0 to 8% did not affect the weights of the breast, thigh, and organs and meat quality, including the meat color, drip loss, cooking loss, and shear force. However, Ramos-Elorduy et al. [14] observed that increasing the inclusion level of *T. molitor* larvae meal from 0 to 10% in diets fed to broiler chickens (7 to 21 day) did not affect the growth performance of broiler chickens. Similarly, Biasato et al. [86] reported that the inclusion of 7.5% *T. molitor* larvae meal in diets fed to broiler chickens (43 to 97 day) had no significant effects on the growth performance, carcass traits, and intestinal morphology. Other studies showed that the inclusion of 29.5% or 29.65% *T. molitor* larvae in diets fed to broiler chickens (30 to 62 day) did not affect the BW and BWG, but decreased FCR [16,40]. Bovera et al. [40] reported that the inclusion of 29.5% *T. molitor* larvae with complete replacement of SBM in broiler diets increased the protein efficiency ratio of broiler chickens. However, Bovera et al. [16] found that the inclusion of 29.65% *T. molitor* larvae with complete replacement of SBM in broiler diets did not affect the carcass yield, but decreased the ileal digestibility of dry matter, organic matter, and crude protein compared to those for the SBM-containing diet. Moreover, Biasato et al. [87] reported that a high inclusion of full-fat *T. molitor* larvae meal from 10 to 15% reduced mucin synthesis and decreased *Firmicutes* and the *Firmicutes/Bacteroidetes* ratio in the cecal microbiota of broiler chickens. The different effects of the inclusion of *T. molitor* larvae in broiler diets in previous studies could partly be attributed to differences in the inclusion level, nutritional value of *T. molitor* larvae, breeder, and age. Therefore, *T. molitor* larvae could be added up to a level of 10% in broiler diets, without having a negative effect on the growth performance and carcass traits. Furthermore, *T. molitor* larvae could completely replace the SBM in broiler diets, without any detrimental impacts.

The apparent ileal digestibility coefficients (AIDCs) of amino acids (AAs) for *T. molitor* larvae were reported in the study of De Marco et al. [15], who observed that the AIDC of all the indispensable AAs in *T. molitor* larvae meal was greater than 0.80. The Thr (0.80) and Met (0.80) for *T. molitor* larvae meal were the least digested indispensable AAs, while the most digestible indispensable AAs were Phe (0.91), Ala (0.90), His (0.85), and Lys (0.85). De Marco et al. [15] indicated that the content and the AIDC of Lys for *T. molitor* larvae meal were similar to those of the SBM, but the content of Met was higher and the AIDC of Met was lower than that of SBM. It should be noted that insect meal as a protein source is more similar to an animal protein source than a plant origin protein source. In the study of De Marco et al. [15], the AIDC of *T. molitor* larvae meal was similar or slightly higher than that of fishmeal for most of the amino acids reported by Ravindran et al. [88], even though the AA content of *T. molitor* larvae meal was lower than that of fishmeal. Furthermore, the AIDC of the dispensable amino acids for *T. molitor* larvae meal was higher than those for SBM and other protein sources, such as full-fat SBM, and sunflower meal [88,89,90]. The standardized ileal digestibility coefficients (SIDCs) of AAs for *T. molitor* larvae meal were reported in the study of Nascimento Filho et al. [91], who observed that the SIDC of all the indispensable AAs in *T. molitor* larvae meal was greater than 0.81. The Thr (0.82) and His (0.81) for *T. molitor* larvae meal had the lowest SIDC among the indispensable AAs, while the Arg (0.92), Phe (0.90), and Lys (0.89) had the highest SIDC among the indispensable AAs. They indicated that the SIDC of Lys, Met, Thr, and Val, which are considered to be the main limiting amino acids for broiler chickens, for *T. molitor* larvae meal were comparable to those for SBM and fishmeal. Therefore, *T. molitor* larvae could be considered as a reasonable protein source for broiler diets due to the good AA content, AIDC, and SIDC values of amino acids.

### 5.2. Swine

Studies of *T. molitor* larvae or larvae meal supplementation in swine diets are relatively less abundant compared to studies on broiler chickens. This might be because a large number of *T. molitor* larvae are needed for supplementation in swine diets, since the feed intake of pigs is greater than that of poultry. The effects of *T. molitor* larvae supplementation in swine diets are presented in Table 6. Increasing the inclusion of *T. molitor* larvae from 0% to 6% in diets formulated with similar metabolizable energy (ME), CP, total Lys, and Met contents increased the BW, average daily gain (ADG), average daily feed intake (ADFI), and gain to feed ratio (G:F ratio) of weaning pigs (0 to 5 weeks after weaning) [18]. The authors explained that the flavor of *T. molitor* larvae improved the palatability of diets, thus increasing the feed intake of weaning pigs. Additionally, increasing the inclusion level of *T. molitor* larvae linearly increased the apparent total tract digestibility (ATTD) of DM, CP, and ash. Furthermore, increasing the levels of *T. molitor* larvae supplementation linearly decreased blood urea nitrogen, which is an indicator of the protein property and amino acid availability for animals, and linearly increased serum insulin growth factor-1 (IGF-1) in weaning pigs at 5 weeks. These results imply that the good protein quality of *T. molitor* larvae improved the CP digestibility and protein availability, resulting in a better growth performance of weaning pigs. Meyer et al. [92] reported that the inclusion of *T. molitor* larvae meal in weaning pig’s diet up to 10% resulted in no difference in the BW, ADFI, and G:F ratio of weaning pigs, and increased the plasma concentrations of Ala, Cit, Glu, Pro, Ser, Tyr, and Val. They also observed that increasing the inclusion level of *T. molitor* larvae meal from 0% to 10% did not affect the plasma concentrations for major carnitine/acylcarnitine species, circulating bile acid species, and lipidomic species. Some studies demonstrated that fishmeal in the diet for weaning pigs could be replaced with *T. molitor* larvae or larvae meal. Ao et al. [66] reported that the supplementation of *T. molitor* larvae at 2% with 100% replacement of fishmeal in weaning pig’s diet resulted in no difference in the growth performance and ATTD of DM, nitrogen (N), and gross energy (GE). They also reported that the replacement of fishmeal with *T. molitor* larvae at 2% did not affect the blood urea nitrogen and serum IgG and IGF. Similarly, Ko et al. [53] reported that the supplementation of defatted *T. molitor* larvae meal, which contained 6% crude fat and 74% crude protein, at 5% for 0 to 14 d and 3% for 14 to 28 d, with 100% replacement of fishmeal (5% during d 0 to 14 and 3% during d 14 to 28) in weaning pig’s diet, resulted in no difference in the growth performance, ATTD of DM, CP, GE, and gut histology of the small intestine in weaning pigs. They also observed that 100% replacement of fishmeal with defatted *T. molitor* larvae meal increased serum IgG at d 14. So far, there has been no published study which was conducted to evaluate the effects of *T. molitor* larvae supplementation on the diets for growing to finishing pigs. Due to the price and supply of *T. molitor* larvae, and higher amount of feed intake in growing to finishing pigs, studies on growing to finishing pigs assessing the optimal supplementation level of *T. molitor* larvae are limited, so there is a need to conduct further study for growing to finishing pigs with the development of the production and supply of *T. molitor* larvae.

The apparent ileal digestibility (AID) of Lys, His, Arg, and Cys was greater in growing pigs fed the diet with 9.95% *T. molitor* larvae than in pigs fed the diet with 9.95% fishmeal [19]. In the study of Yoo et al. [19], the standardized ileal digestibility (SID) of Arg and Cys was even greater in pigs fed the diet with 9.95% *T. molitor* larvae than in pigs fed the diet with 9.95% fishmeal. Cho et al. [20] reported that the AID values of Lys, Met, and Thr were similar in pigs fed 10% defatted *T. molitor* larvae meal and 10% *T. molitor* larvae hydrolysate containing diets, but the AID values of Lys, Met, and Thr for defatted *T. molitor* larvae meal and *T. molitor* larvae hydrolysate were greater than those in pigs fed 10% fermented poultry by-product- or 10% hydrolyzed fish soluble-containing diets. For the SIDs of Lys, Met, and Thr, the pigs fed *T. molitor* larvae hydrolysate- and defatted *T. molitor* larvae meal-containing diets showed greater SIDs than the pigs fed fermented poultry by-product- and hydrolyzed fish soluble-containing diets. These observations indicated that 10% *T. molitor* larvae as a protein source resulted in a greater AA digestibility than conventional animal proteins, including fishmeal, poultry meal, and meat meal. Therefore, with regards to the results of previous studies, *T. molitor* larvae could be used as a protein source at a level of up to 6% for weaning pigs and 10% for growing pigs.

## 6. Challenges in the Use of *Tenebrio molitor* Larvae

### 6.1. Safety

Using the insects in animal feed should consider the safety because insects contain chemical defense substances as a toxin produced by the exocrine gland [93]. Additionally, *T. molitor* can contain benzoquinone compounds as a toxin that is secreted by the defensive gland [94]. The benzoquinone is a toxic metabolite for humans and animals, which can interfere with cellular respiration and resulted in kidney damage, as well as have a carcinogenic effect [95]. Generally, the concentration of benzoquinone is accumulated continuously, so it can be increased as the age of *T. molitor* increases. However, so far, it has not been clearly established how much benzoquinone remains in the *T. molitor* larvae after processing methods, including cleaning, drying, heating, and grinding, and what tolerant levels of benzoquinone in monogastric animals are. Therefore, there is a need to establish a processing method to control the toxicity or residual amount of the benzoquinone in *T. molitor* larvae products.

Insects may have antibiotic resistance genes [96], implying that insects can be contaminated by pathogens or mycotoxin from contaminated diets. Wynants et al. [97] observed that contaminated wheat bran caused the *Salmonella* spp. in *T. molitor* larvae. However, regular microbial monitoring on pathogens of the substrate and the larvae can prevent the survival of pathogens in the *T. molitor*. Ravzanaadii et al. [62] demonstrated that there was no detection of *Escherichia coli* and *Salmonella* spp. in *T. molitor* larvae and adults fed wheat bran and vegetables such as cabbage, reddish, and carrot. Interestingly, *T. molitor* larvae fed the diets contaminated with the mycotoxin deoxynivalenol grew normally and degraded the mycotoxin [98]. Camenzuli et al. [99] reported that the *T. molitor* larvae fed the diet with aflatoxin B_1_, deoxynivalenol, ochratoxin A, or zearalenone did not show any mycotoxin accumulation in their body. However, since it is not clear how *T. molitor* larvae deal with the mycotoxins, further research is needed.

Insects can accumulate heavy metals in their tissue from the rearing environment and their feed [100,101]. For instance, feed ingredients such as wheat, rice, mussels, and animal kidney corns were shown to contain a high level of cadmium [102]. Mlček et al. [103] reported that the accumulated heavy metal content is dependent on the feed and that the level of Pb in *T. molitor* larvae was below the detection limit, but the content of Cd in the dry matter of *T. molitor* larvae (147 to 230 mg/kg) was above the food limit. The accumulation of heavy metals in insects may be toxic to animals and humans. Therefore, heavy metal screening such as X-ray fluorescence spectrometry should be employed to detect heavy metals before using *T. molitor* larvae as a feed ingredient.

### 6.2. Consumer Acceptance

The consumer’s acceptance of meat products from animals fed insects should be also considered. A few studies surveyed the consumer’s willingness to buy animal products that originated from animals fed insects as feed [104,105,106]. Those studies observed that the acceptance rates of meat products from animals fed insects (e.g., fish, poultry, pigs, and cattle) were above average. Additionally, the consumer seems to prefer meat products from fish fed insects rather than from poultry, pigs, and cattle fed insects as feed [104,105]. This implies that the effort of improving the consumer’s perspective toward meat products from animals fed insects is needed. If comparing the preference for poultry and pigs, Verbeke et al. [104] observed that the attitudes toward the use of insects in poultry feed are higher than in pigs. As both poultry and pigs are omnivorous animals, consuming insects is natural behavior. Therefore, providing information about the meat composition from animals fed insects is recommended to alter the consumer’s perspective.

### 6.3. Price

To use *T. molitor* larvae in the diets of monogastric animals, the stable production and a competitive price for *T. molitor* larvae should be ensure first. Up till now producers that supply insects (including *T. molitor* larvae) have a small sized production facility and low production and efficiency because they supply the *T. molitor* larvae as food for birds and reptiles, with a relatively small market. In this situation, the supply quantity and price for *T. molitor* larvae are less competitive than those for SBM and fishmeal, which are widely used in poultry or swine diets as a protein source. In 2020, the market price of *T. molitor* larvae (mealworm) being retailed varies in each country. The market price for *T. molitor* larvae is 8.4 to 9.3 $/kg in China, 10.8 to 14 $/kg in the USA, 12.9 to 20 $/kg in the EU, and 65 to 70 $/kg in South Korea, which are considerably higher than the price of fishmeal of 1.2 to 1.3 $/kg and SBM of 0.34 $/kg. With the increasing interest and demand for insects as a future food resource, continuous investment and technological development in the insect industry are being made, so stable mass production and reasonable prices will be obtained in the future.

## 7. Conclusions

*T. molitor* larvae show significant potential to be used as a protein source in poultry and swine diets. Some commercially produced *T. molitor* larvae or protein concentrate products have been approved for use as protein supplements in certain countries. Several studies have been conducted for evaluating the use of *T. molitor* larvae in monogastric animal diets. The effects of the supplementation of *T. molitor* larvae on broiler chickens or pigs and the optimal inclusion level of *T. molitor* larvae in the diets of monogastric animals can be attributed to the nutritional value of *T. molitor* larvae and the age or species of monogastric animals. However, the use of *T. molitor* is still limited in controlling toxin and heavy metals from *T. molitor*. Moreover, the consumer’s acceptance of the meat products from monogastric animals fed insects and the price of *T. molitor* larvae are challenges that need to be overcome in order to use *T. molitor* larvae in the feed as an alternative protein source. Therefore, *T. molitor* larvae could be used as an alternative or sustainable protein source in monogastric animal feed with a consideration of the nutritional values, biosafety, consumer acceptance, and market price of such products.

## Figures and Tables

**Table 1:** Nutritional value of *Tenebrio molitor* larvae or larvae meal (DM basis).

	*T. molitor* Larvae Meal			*T. molitor* Larvae									Conventional SBM	Fishmeal
Crude protein, % ^1^ (nitrogen-to-protein conversion factor *k_p_*)	55.27 (6.25)	55.30	53.83	47.70	50.79 (6.25)	47.00	53.22 (6.25)	46.44	60.21	58.00	50.96 (5.41)	48.33	49.44	67.53
Corrected crude protein, % ^2^	47.84				43.96		46.07							
Crude fat, %	29.54	22.97	28.03	37.70	36.77	29.60	34.54	32.70	19.12	31.6		36.06	1.40	10.36
Ash, %		4.99	6.99		6.70	2.56	4.04	2.86	4.20	3.00		2.65	7.19	17.15
Crude fiber, %			7.53	5.00	6.48	5.60	6.26	4.58	22.35	4.90		4.19	7.43	0.26
Acid detergent fiber, %		7.66											7.50	
Chitin, %			5.60			8.91					4.30			
References	[15]	[16]	[75]	[14]	[19]	[28]	[54]	[62]	[63]	[64]	[65]	[76]	[61]	[61]

^1^ Published data; ^2^ Corrected value of crude protein with the nitrogen-to-protein conversion factor *k_p_* = 5.41 of Boulos et al. [65]. DM: dry matter, SBM: soybean meal.

**Table 2:** Amino acid composition of *Tenebrio molitor* larvae or larvae meal (DM basis).

	*T. molitor* Larvae Meal		*T. molitor* Larvae								Conventional SBM	Fishmeal
Indispensable amino acids, %												
Arginine	2.80	3.84	2.03	5.80	2.23	2.43	2.23	4.42	2.21	1.89	3.57	4.10
Histidine	1.68	2.25	1.07	3.11	2.80	1.53	1.38	2.77	1.65	0.84	1.42	1.54
Isoleucine	2.21	2.80	1.39	4.00	1.98	3.56	1.83	6.48	4.51	1.31	2.21	2.73
Leucine	3.15	4.81	2.81	7.31	3.37	3.41	3.13	6.21	5.32	2.21	3.86	4.77
Lysine	3.59	1.79	1.86	5.76	2.01	2.91	2.50	5.31	4.51	1.58	3.11	4.87
Methionine	1.01	1.43	0.54	2.20		0.67	0.52	1.22	1.34	0.60	0.68	1.85
Phenylalanine	1.88		1.36	3.95	1.76	1.76	1.55	3.20	1.54	1.31	2.55	2.64
Threonine	1.85	2.89	1.57	4.29	1.83	1.81	1.70	3.31	1.64	1.27	1.98	2.75
Valine	2.82	3.96	3.14	5.29	2.94	2.44	2.57	4.46	4.42	1.89	2.17	3.27
Tryptophan		1.86		0.65				0.02		0.30	0.66	0.67
Dispensable amino acids, %												
Alanine	3.89		3.15	7.46	3.96	3.69		6.70	4.34	2.48	2.16	4.19
Aspartic acid	4.37		3.07	8.51	2.76	3.59		6.52	3.23	1.54	5.50	5.77
Cysteine	1.25		0.35		3.16	0.52		0.93		1.19	0.77	0.65
Glycine	2.21		2.04	5.38	2.61	2.41		4.38	2.65	1.71	2.13	5.03
Glutamic acid	6.29		4.57	12.26	5.78	5.68		10.32	4.75	3.92	8.86	8.41
Proline	3.43		2.23	7.15	1.66	3.02		5.52	2.34	2.00	2.74	3.08
Serine	2.27		1.86	5.13	2.20	2.09	2.23	3.82	3.45	1.36	2.41	2.59
Tyrosine	3.28		2.63	8.25	3.45	3.46		6.32	2.32	2.15	1.55	2.01
References	[15]	[16]	[18]	[19]	[54]	[62]	[63]	[64]	[76]	[77]	[61]	[61]

DM: dry matter, SBM: soybean meal.

**Table 3:** Fatty acid composition of *Tenebrio molitor* larvae (DM basis).

	*T. molitor* Larvae				
Fatty acids, %					
Myristic acid (C14:0)	2.85	5.21	3.05	3.26	2.12
Pentadecanoic acid (C15:0)	7.10	0.06			0.22
Palmitic acid (C16:0)	9.33	15.05	16.72	17.21	17.24
Palmitoleic acid (C16:1)	2.12	2.84	2.67		1.94
Stearic acid (C18:0)	2.40	0.26	2.49	3.06	0.69
Oleic acid (C18:1n9)	40.78	49.71	43.17	44.36	43.77
Linoleic acid (C18:2n6)	35.58	24.19	30.23	31.63	29.39
Linolenic acid (C18:3n3)		0.35	1.36	1.46	2.27
γ-Linoleic acid (C18:3n6)	1.85	0.03	0.05		
Eicosenoic acid (C20:1n9)		0.06	0.24	0.39	
Arachidonic acid (C20:4n6)				0.50	
Erucic acid (C22:1)					1.62
Docosatetraenoic acid (C22:4n6)		0.13		0.41	
Saturated fatty acid, %		22.17	22.26	23.34	20.99
Unsaturated fatty acid, %		77.83	77.74	78.41	79.01
UFA/SFA ratio		3.51	3.49	3.60	3.76
n-6/n-3(omega 6/omega 3) ratio		69.73	0.69		12.98
References	[18]	[54]	[62]	[63]	[77]

DM: dry matter, UFA: unsaturated fatty acid, SFA: saturated fatty acid.

**Table 4:** The mineral contents of *Tenebrio molitor* larvae (DM basis).

	*T. molitor* Larvae			
Calcium, %	0.08	0.04	0.50	0.38
Phosphorus, %	1.04	0.71	0.98	0.70
Sodium, %	0.11	0.36		
Potassium, %	0.74	0.95	0.95	0.85
Magnesium, %	0.32	0.20	1.63	
Iron (Fe), mg/kg	100.02	66.87	68.2	63.0
Zinc (Zn), mg/kg	117.40	104.28	106.0	102.0
Copper (Cu), mg/kg	20.00	13.27	19.0	12.3
References	[54]	[62]	[63]	[65]

DM: dry matter.

**Table 5:** Effects of *Tenebrio molitor* larvae or larvae meal supplementation on broiler chickens.

References	Breed	Ingredient Type	Supplementation Level, %	Age	Performance
[14]	Arbor Acers × Vantress broiler chickens (male and female)	*T. molitor* larvae meal	0, 5, 10	7–21 d	No difference in BWG, FI, and FCR
[16]	Shaver brown broiler (male)	*T. molitor* larvae meal	0, 29.65	30–62 d	No difference in BW and BWG Decrease FCR No difference in carcass yield Decrease ileal digestibility of DM, OM, and CP
[17]	Ross 708 (male)	Full-fat *T. molitor* larvae meal	0, 5, 10, 15	0–53 d	Increase BW at 12 and 25 d Increase daily FI, and FCR No difference in weights of carcass, breast, and thigh
[28]	Ross 308 (female)	Full-fat *T. molito*r larvae meal	0, 0.2, 0.3	0–35 d	Increase BWG and FI Decrease bursa of Fabricius weight No difference in blood total protein and albumin. Increase serum IL-2 and TNF-α
[28]	Ross 308 (female)	Full-fat *T. molito*r larvae meal	0, 0.3	0–35 d	Increase FI and FCR Decrease blood non-esterified fatty acid Decrease serum IgM No difference in serum IgY, IgA, IL-2, IL-6, and TNF-α
[40]	Shaver brown broiler (male)	*T. molitor* larvae	0, 29.5	30–62 d	No difference in BW and BWG Decrease FCR Increase protein efficiency ratio
[75]	Arbor Acres	*T. molitor* larvae meal	0, 2.5, 5	0–25 d	Increase BWG for 0–10 d No difference in FI and FCR No difference in mortality, carcass yield, and organ weights
[76]	Broiler chickens from commercial hatchery	*T. molitor* larvae	0, 0.1, 0.2, 0.3	0–42 d	Increase BWG Decrease FCR Increase dressing rate
[85]	Ross 308 (male)	*T. molitor* larvae meal	0, 2, 4, 8	0–42 d	Increase BW, ADG, and FCR for 0–21 d No difference in weights of carcass, breast, thigh, and organs. No difference in meat quality (color, drip loss, cooking loss, and shear force)
[86]	Label Hubbard hybrid chickens (female)	*T. molitor* larvae meal	0, 7.5	43–97 d	No difference in growth performance, carcass traits, and intestinal morphology
[87]	Ross 708 (male)	Full-fat *T. molitor* larvae meal	0, 5, 10, 15	0–40 d	Decrease *Firmicutes* and *Firmicutes*/*Bacteroidetes* ratio in cecal microbiota (*T. molitor* larvae meal 10–15%) Decrease mucin synthesis (*T. molitor* larvae meal 10–15%)

ADG: average daily gain, BWG: body weight gain, CP: crude protein, DM: dry matter, FCR: feed conversion ratio, FI: feed intake, Ig: immunoglobulin, IL: interleukin, OM: organic matter, TNF: tumor necrosis factor. d: day.

**Table 6:** Effects of *Tenebrio molitor* larvae or larvae meal supplementation on pigs.

References	Phase	Breed	Sex	Ingredient Type	Supplementation Level, %	Age	Performance
[18]	Weaning pigs	(Yorkshire × Landrace) × Duroc	Gilt and barrow	*T. molitor* larvae	0, 1.5, 3.0, 4.5, 6.0	0–35 d after weaning	Increase BW, ADG, ADFI, and G:F ratio Decrease BUN Increase IGF-1 at 35 d Increase the ATTD of DM, and CP
[53]	Weaning pigs	Landrace × Yorkshire × Duroc		Defatted *T. molitor* meal	5–3, 2.5–1.5	0–28 d after weaning	No difference in growth performance No difference in ATTD of DM, GE, and CP Increase serum IgG at 14 d No difference in gut histology for duodenum, jejunum, and ileum
[66]	Weaning pigs	(Yorkshire × Landrace) × Duroc (gilt and barrow)	Gilt and barrow	*T. molitor* larvae	0, 2	0–35 d after weaning	No difference in growth performance No difference in ATTD of DM, N, and GE No difference in serum IgG, IGF, and BUN
[92]	Weaning pigs	(German Landrace × German Edelschwein) × Peitrain	Male	*T. molitor* larvae meal	0, 5, 10	0–28 d after 5 week old	No difference in final BW, ADFI, and G:F ratio Increase plasma Ala, Cit, Glu, Pro, Ser, Tyr, and Val No difference in plasma concentration of major carnitine/acylcarnitine species and circulating bile acid species No difference in plasma triglyceride, cholesterol, and phospholipid

ADFI: average daily feed intake, ADG: average daily gain, ATTD: apparent total tract digestibility, BUN: blood urea nitrogen, BW: body weight, CP: crude protein, DM: dry matter, G:F ratio: gain to feed ratio, GE: gross energy, IGF: insulin-like growth factor, IgG: immunoglobulin G, N: nitrogen. d: day.
